# MathIOmica‐MSViewer: a dynamic viewer for mass spectrometry files for Mathematica

**DOI:** 10.1002/jms.3928

**Published:** 2017-05-08

**Authors:** R. Roushangar, G. I. Mias

**Affiliations:** ^1^ Department of Biochemistry and Molecular BiologyMichigan State UniversityEast LansingMI48824USA

**Keywords:** mass spectrometry, bioinformatics, data mining, Mathematica

## Abstract

MathIOmica‐MSViewer is an add‐on graphical user interface utility for the Mathematica software system which facilitates the visualization and exploration of spectra from open format mass spectrometry files (mzXML and mzML standard community formats). The viewer was designed for simplicity and handling of large mass spectrometry data files. To facilitate searches, users may use search filters for the spectra based on mass to charge ratios and retention times, and visualize precursor spectra associated to a parent spectrum. *Availability:* The viewer is available as a Mathematica notebook (MathIOmica‐MSViewer.nb) at https://doi.org/10.5281/zenodo.321385. The software is provided under an MIT License. © 2017 The Authors. *Journal of Mass Spectrometry* published by John Wiley & Sons, Ltd.

## Introduction

The rapid advancements of mass spectrometry have led to the increased availability of mass spectrometry data, especially in the fields of proteomics and metabolomics. A typical mass spectrometry experiment generates multiple data as intensity spectra, measuring the different levels of ion fragments as a function of their mass to charge ratio (*m/z*). These spectra, along with information about experimental conditions, additional information such as chromatography results and metadata regarding the spectrometers, are typically stored in files of proprietary formats specific to each hardware/spectrometer vendor.^1^ These data must then undergo extensive analysis depending on the experiment, to enable identification of the ions as peptides/proteins or molecules, and quantification, towards experimental interpretation. Vendors typically provide their own software for such an analysis. However, this makes it difficult to use, develop and test more analysis algorithms, and open source software, and importantly to share results with the scientific community.

Many open file formats emerged by the mid‐2000s to address these concerns,^1^ with the most popular being mzData, developed by the Human Proteome Organization‐Proteomics Standards Initiative (HUPO‐PSI) as a common standard, and mzXML, developed by the Institute for Systems Biology as a format aimed at an analysis platform that could be used across multiple vendor results.^2^, ^3^ Both formats were based on eXtensible Markup Language (XML), which enables textual descriptions and metadata to be included along with numerical data information. While straightforward to use, having two formats created an unnecessary extra burden for developers building software that needed to support the analysis of results from both file formats. A collaboration between the HUPO‐PSI and Institute for Systems Biology incorporated elements of both formats and eventually developed a new community simple standard, mzML.^1^, ^2^, ^4^, ^5^

Raw data are now being converted routinely from vendor‐proprietary formats, to the open standards discussed above, particularly mzXML and now mzML (for example by using programs as MSConvert^6^). In downstream applications, the spectra can then be annotated and features identified: For example, in proteomics, the mass spectra can then be mapped to proteins, using multiple software such as X!Tandem,^7^ Open Mass Spectrometry Search Algorithm (OMSSA),^8^ Mascot,^9^ Proteome Discoverer by Thermo Scientific and the SEQUEST algorithm^10^ and MassHunter Workstation by Agilent Technologies. Downstream analysis can also be carried out using suites and programs, such as the Trans‐Proteomic Pipeline (TPP),^11^ ProteoWizard,^6^ PEAKS^12^ and many others. Similarly, in metabolomics, the spectral data are typically aligned for retention time and mass intensity calibration, using software such as the widely used XCMS,^13^ MzMine,^14^ various MATLAB (TheMathWorks) toolboxes and MassHunterProfiler (Agilent). A frequently updated list of various programs for mass spectrometry is also available online.^14^

In routine experiments, and following data acquisition, it is highly useful to be able to inspect the raw spectra prior to conducting extensive analysis. Additionally, the continuous improvements in mass spectrometry instrumentation have led to more precise experiments with more data, i.e. more spectra per experiment acquired at higher resolutions. This in turn has resulted in larger mass spectrometry files being generated, which are more cumbersome to navigate, and viewing raw data through loading entire files into search engines can become time consuming, further highlighting the need for a simple viewer for a first pass at the data. There are various viewers that have become available, which also involve some form of data processing, including MZMine2,^15^ Mass++,^16^ TOPPView^17^ and mzR,^18^ but while these greatly facilitate analysis, they are not dedicated to simple data inspection. There are also few dedicated viewers, written in various programming languages: omniSpect,^19^ for MATLAB, mMass3,^20^ written in Python, MS‐Viewer,^21^ which is web based, jmzReader,^22^ which offers a Java library for parsing spectra files, and BatMass,^23^ which presents an updated Java implementation and interface, and is probably the most efficient of the above, in terms of quickly parsing large files.^23^ To the best of our knowledge, no standalone versions exist in the Wolfram Language, which is the coding language used in the Mathematica^24^ software, besides embedded functionality in the program MathDAMP,^25^ originally aimed at metabolomics analysis.

In this Application Note, we describe the release of MathIOmica‐MSViewer, a mass spectrometry spectral viewer designed for simplicity, stability and speed, and distributed as an standalone add‐on to the MathIOmica^26^ package^27^ for Mathematica. The software is aimed at experimental mass spectrometry researchers interested in accessing and visually inspecting raw data spectra from standard open formats mzML and mzXML, prior to further data analysis. It is offered as a supplement to existing viewers, extending the existing repertoire to users of Mathematica and the Wolfram Language.

## Methods

**Figure 1:**
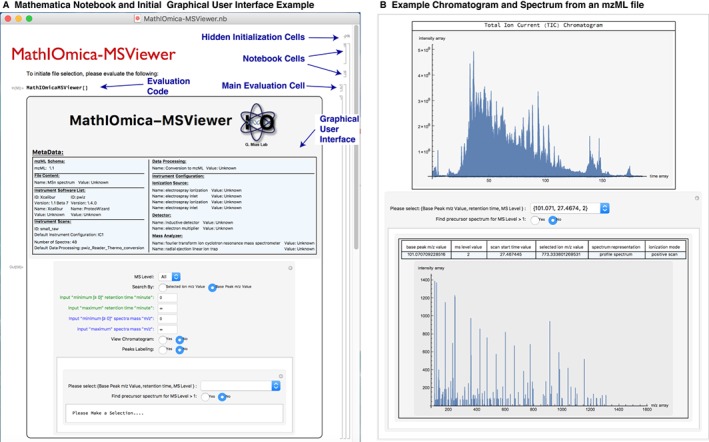
MathIOmica‐MSViewer implementation. The MathIOmica‐MSViewer viewer is deployed in a Mathematica file, termed a notebook, A. Both code and results in a notebook are grouped in paragraphs called cells. The spectral viewer output utilizes a responsive graphical user interface. Once the user identifies the file to import from a pop‐up selection, they are presented with the metadata information and filtering window. The selections allow the user to specify either the base peak *m/z* value, or the selected ion *m/z*. The resulting spectrum with summary information is then plotted as in the example B.

The MathIOmica‐MSViewer is deployed as a Mathematica (Wolfram Research) notebook file. Such notebook files (with extension ‘nb’) act as the main user interface for Mathematica (Fig. 1A). Notebook files utilize a text‐based content, which is used interactively to present both Wolfram Language code and display the corresponding calculation results concurrently, including graphical output in Mathematica. The input and output lines in any notebook are grouped in paragraphs, called cells, which are outlined by square brackets (Fig. 1A). The MathIOmica‐MSViewer.nb notebook includes all the code (as Wolfram Language function definitions) necessary to run the mass spectrometry viewer, has a small file size footprint (~2 MB) and provides a graphical user interface to search, and visualize basic mass spectrometry spectra from an input file (Fig. 1B). The notebook is platform independent and has been tested with Mathematica version 10.3+ on Windows (versions 8 and higher), Linux (Ubuntu versions 14+) and Mac OS (versions 10.9+). In terms of hardware requirements, the software will run on any system able to run Mathematica 10.3+, with a recommended minimum of 2 GB of RAM installed.

**Figure 2:**
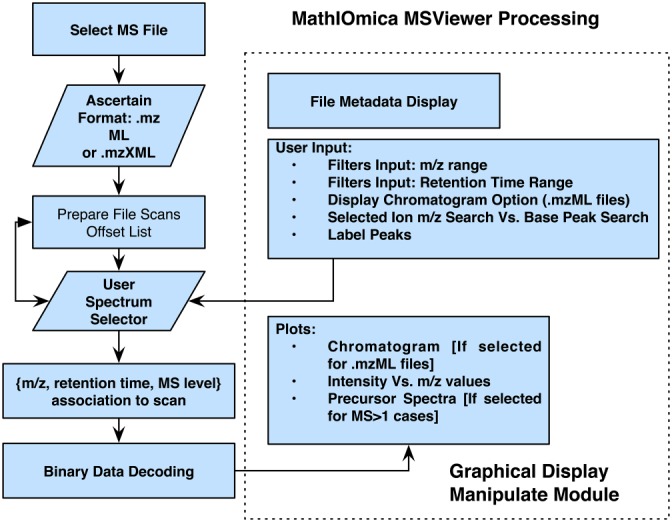
MathIOmica‐MSViewer workflow. The workflow summarizes the internal functionality and information flow between the graphical user interface and background processes. [Colour figure can be viewed at wileyonlinelibrary.com]

The MathIOmica‐MSViewer.nb notebook (Fig. 1A) contains initialization cells, which are cells that contain code definitions hidden from the main view that are evaluated automatically to declare (initialize) all necessary code in the background at the loading of the program. The main evaluation code text, ‘MathIOmicaMSViewer[ ]’ is presented in a cell (Fig. 1A) and is automatically evaluated on opening the notebook file. Alternatively, the user may re‐evaluate the cell at any time by placing a cursor on the main evaluation cell (Fig. 1A) and pressing the shift and enter‐buttons simultaneously (this is the standard way of evaluating code in Mathematica). Upon evaluation, a graphical system file browser/selector directs the user to select the files to be viewed (mzML or mzXML formats are acceptable). Once file selection is completed, the file format is internally ascertained, and an internal indexing of spectral scans is performed (Fig. 2). This indexing is used to avoid loading the entire input file to memory. The scan information for each spectrum can then be streamed in as necessary based on the user's subsequent selections, which allows for a smaller memory footprint by loading one scan at a time, as well as fast access/selection of spectra to plot from large data files.

In terms of the internal code, a Wolfram Language ‘Manipulate’ function has been used to create the responsive graphical user interface (Fig. 2). In the background, XML is parsed internally for all the information that is then formatted for the graphical user interface. First, the metadata contained in the selected mass spectrometry file are displayed. For mzML files, these metadata are internally annotated using derived controlled vocabularies, which have been compiled using the standards provided by the HUPO‐PSI Mass Spectrometry Standards Working Group, (https://github.com/HUPO‐PSI/psi‐ms‐CV/blob/master/psi‐ms.obo, v4.0.3). In the case of mzXML files, the metadata descriptions are read in directly from the XML.

After the input file is loaded and the index is created, the user is provided access to available scans through a drop‐down menu in the lower part of the interface (Fig. 1A). These scan options displayed in the dropdown menu will depend on the user's search filtering selections: User search filters can implement a narrow selection by *m/z* values (for either base peak, or selected ion masses for MSn scans), and retention times. Once search filters are finalized, a selection can be made from the dropdown menu, which will result in the display of the associated spectra in the viewer window. Internally these spectra are obtained by decoding the binary structures in the XML file and uncompressing the data (Fig. 2).

Additionally, the interface provides check‐boxes for a few additional options: First, the checked option to label peaks allows the coordinates of any peak to be interactively displayed, as the pointer is located at the peak of interest. If other scans need to be selected for visualization, the user can choose new selection filters to aid the selection from the dropdown menu. Second, there is a checked option to visualize chromatograms for mzML files, which are displayed above the associated spectra. Third, for MSn scans, the user is provided with a checked option to choose whether to view all the precursor spectra for a selected scan.

The software has been tested on both mzML and mzXML files, with multiple MS levels, using public and in‐house datasets up to 7 GB, including decoding spectral data that were encoded for compression in both the binary and zlib‐compressed binary data formats that are used in mzML and mzXML files.

## Summary

We have created an easy to use utility (MathIOmica‐MSViewer) using a Mathematica notebook file format that enables searching, parsing and visualizing mass spectrometry spectra, from standard mzML and mzXML formatted files. The MathIOmica‐MSViewer has been designed for simplicity and quick access of spectra in large files, providing a graphical user interface with search filtering capabilities. While we anticipate additional functionality to be added in the future in more extended applications, our plan is to also maintain this version of the viewer for its speed of application and ease of use.

The latest MathIOmicaMS‐Viewer release is available online, at Zenodo via GitHub at https://doi.org/10.5281/zenodo.321385, as well as backed up at https://mathiomica.org under Extra Tools. The viewer is platform independent with regards to operating system and is written in the Wolfram Language, requiring Mathematica 10.3+ to run. The software is released under an open source MIT License.

## Additional files:

- MathIOmica‐MSViewer_Manual (manual for viewer)
- MathIOmica‐MSViewer.nb (viewer software as Mathematica notebook)
- MathIOmica‐MSViewer_SourceCode (viewer software with source code visible)

## Authors' contributions

G.I.M. conceived of and directed the project, and wrote the manuscript and code. R.R. wrote source code and tested the application, and participated in manuscript preparation.

## Supporting information

Data S1. MathIOmica‐MSViewer Manual
