# Supplementary material for: MathIOmica‐MSViewer: a dynamic viewer for mass spectrometry files for Mathematica
Source: J Mass Spectrom. 2017 May 8;52(5):315–8. doi: 10.1002/jms.3928 (PMC5435938; doi:10.1002/jms.3928)
Supplement: Supplementary file 1 — Data S1. MathIOmica‐MSViewer Manual [file JMS-52-315-s001.zip › MathIOmica-MSViewer_Manual.pdf]

MathIOmica-MSViewer: A Dynamic Viewer for Mass Spectrometry Files for Mathematica

Raeuf Roushangar[1], George I. Mias[1,*].
[1]Department of Biochemistry and Molecular Biology, Michigan State University, East Lansing MI 48824
*To whom correspondence should be addressed.

MathIOmica-MSViewer Manual

Summary

MathIOmica-MSViewer is a standalone mass spectrometry data viewer for Mathematica, with a simple graphical user interface released as an add-on utility for the MathIOmica package. It is provided as a notebook - that may be copied and redistributed, that allows the user to open and view mzML and .mzXML spectra. The notebook is platform independent (can work on Windows, Linux and Mac OS), and has been tested on Mathematica 10.3+.

Initialization and Selecting Files

On opening the MathIOmica-MSViewer.nb notebook the initialization cells will automatically be evaluated. Depending on the settings, you may be prompted to allow the initialization to proceed. Please select "OK" from the popup menu if so asked.

The initial notebook contains the following cell:

```
MathIOmicaMSViewer[]
```

To import data:
1. place your cursor in the cell and press simultaneously the shift+enter (Shift + Return on Mac keyboards) to evaluate it.
2. A selection interface window will open.
3. Navigate to your spectra file (.mzML or .mzXML formats), and select the file by clicking on it.
4. Press "Open".

MathIOmicaMSViewer will open the file for reading and present a summary of metadata:

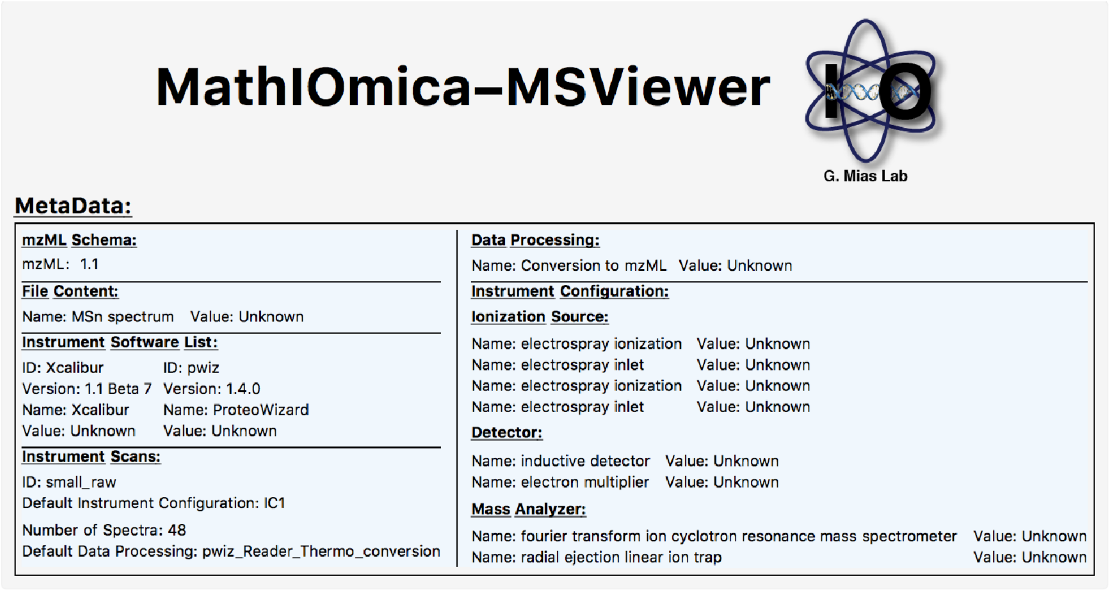

Selecting Spectra for Viewing

After the metadata, the following Manipulate panel is shown:

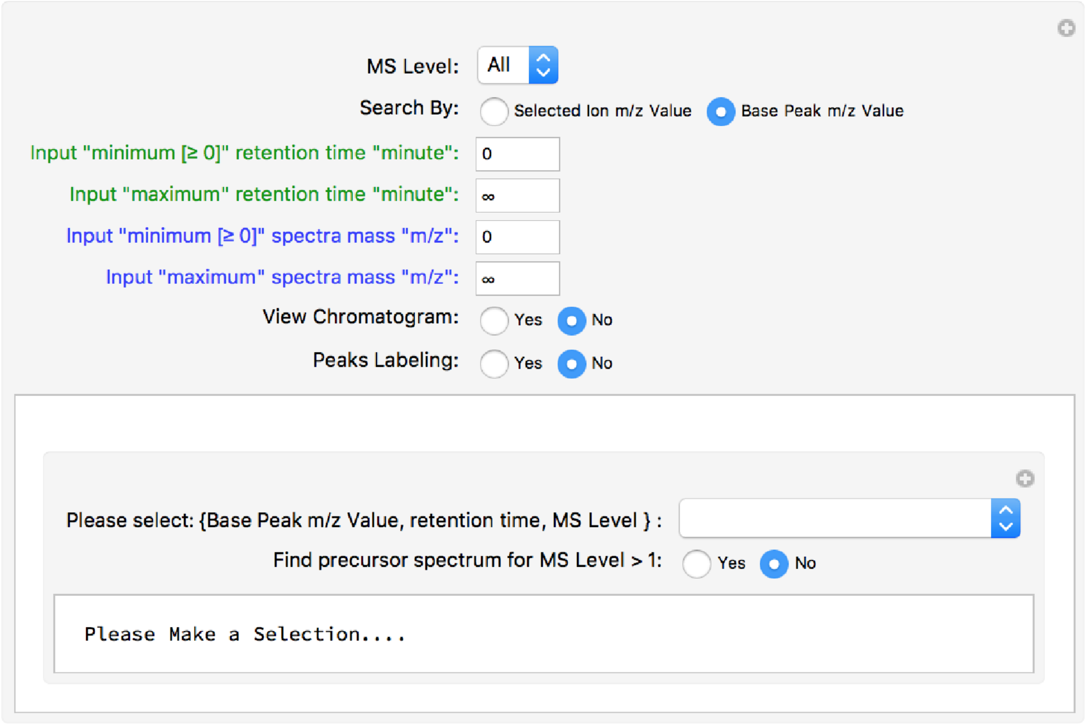

The various selections allow the filtering of spectra to consider based on retention time and mass or m/z ratios reported in the spectra, choices to use Selected Ion m/z values or Base Peak m/z Values in these filters. The choices set the selection availability in the lower panel. First, the following selections are made to narrow down the choices:

Option (Upper Panel)	Selection Choices	Explanation
MS Level	Dropdown Menu: select an integer in {1,2,..., n}	Selection of the level of MS to be viewed. The number corresponds to: 1 : MS, 2 : MS/MS, ..., n : MS^n
Search By:	Radio Button Choices: • Selected ion m/z Value • Base Peak m/z Value	Selection of the type of m/z values in each spectrum to which filtering will be applied. The values to be used for filtering can be either selected ion m/z values (for MS Level⩾2), or base peak m/z values
Input minimum [≥0] retention time	Numeric input greater than zero	Minimum retention time for filtering of spectra to view
Input maximum retention time	Numeric input greater than zero	Maximum retention time for filtering of spectra to view. Must be greater than minimum retention time.
Input minimum [≥0] spectra mass "m/z"	Numeric input greater than zero	Minimum mass to charge ratio used to filter spectra to view.
Input maximum [≥0] spectra mass "m/z"	Numeric input greater than zero	Maximum mass to charge ratio used to filter spectra to view.
View Chromatogram (.mzml files only)	Radio Button: select Yes or No	Selection of whether to view the Chromatogram for the file if available.
Peak Labeling	Radio Button: select Yes or No	Whether to show labels for the peaks when hovering over with the pointer. N.B. For dense spectra this operation may be intensive/time consuming.

Following the selections, the lower panel allows the selection of the available spectra based on the filtering:

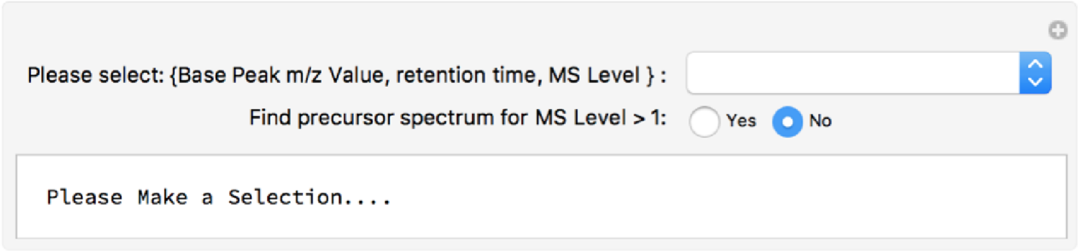

Option (Lower Panel)	Selection Choices	Explanation
Please select {Base Peak (or Selected Ion) m/z Value,retention time,MS Level}	Dropdown Menu	The available choices are the result of the filtering and choices selected in the upper part of the panel
Find precursor spectrum for MS Level >1	Radio Button : select Yes or No	Whether to find and display the precursor spectrum for MS >1 spectra

A Selection from the Dropdown menu will result in the plot of the spectra. For an MS2+ spectrum, if the selection to Find the precursor spectrum was made, the precursor spectrum will be displayed as well:

base peak m/z value	ms level value	scan start time value	selected ion m/z value	spectrum representation	ionization mode
579.15649414⋱0625	2	0.2726633333⋱3333331	725.36000000⋱000001	centroid spectrum	positive scan

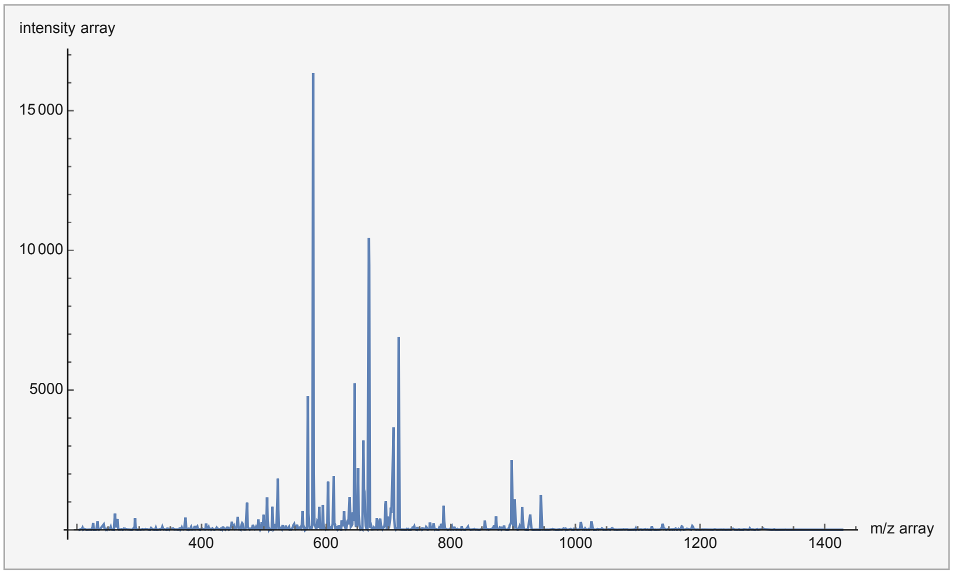

base peak m/z value	ms level value	scan start time value	spectrum representation	ionization mode
811	1	0.216746666666666⋱67	profile spectrum	positive scan

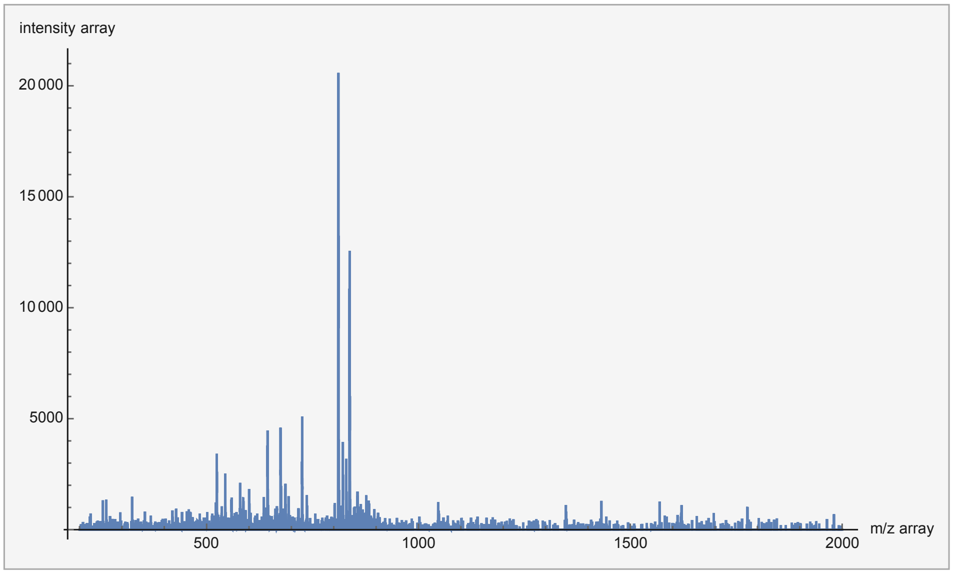

Additional Notes

Initialization Cells

The notebook contains multiple initialization cells hidden from view. These should not be removed as they contain the necessary definitions for the viewer to run.

Licensing

The MIT License (MIT). MathIOmica is a general omics data analysis framework. The MathIOmica-MSViewer tool is provided as a standalone add-on tool to MathIOmica under an MIT License, Copyright (c) 2016 George I.Mias, G.Mias Lab, Department of Biochemistry and Molecular Biology, Michigan State University, East Lansing 48824.
Permission is hereby granted, free of charge, to any person obtaining a copy of this software and associated documentation files (the \" Software \"), to deal in the Software without restriction, including without limitation the rights to use, copy, modify, merge, publish, distribute, sublicense, and/or sell copies of the Software, and to permit persons to whom the Software is furnished to do so, subject to the following conditions : The above copyright notice and this permission notice shall be included in all copies or substantial portions of the Software.THE SOFTWARE IS PROVIDED \" AS IS \", WITHOUT WARRANTY OF ANY KIND, EXPRESS OR IMPLIED, INCLUDING BUT NOT LIMITED TO THE WARRANTIES OF MERCHANTABILITY, FITNESS FOR A PARTICULAR PURPOSE AND NONINFRINGEMENT.IN NO EVENT SHALL THE AUTHORS OR COPYRIGHT HOLDERS BE LIABLE FOR ANY CLAIM, DAMAGES OR OTHER LIABILITY, WHETHER IN AN ACTION OF CONTRACT, TORT OR OTHERWISE, ARISING FROM, OUT OF OR IN CONNECTION WITH THE SOFTWARE OR THE USE OR OTHER DEALINGS IN THE SOFTWARE.
